# A multicenter non-inferior randomized controlled study comparing the efficacy of laparoscopic versus abdominal radical hysterectomy for cervical cancer (stages IB1, IB2, and IIA1): study protocol of the LAUNCH 2 trial

**DOI:** 10.1186/s13063-022-06245-5

**Published:** 2022-04-08

**Authors:** Xin Wu, Ling Qiu, Weihua Lou, Xipeng Wang, Tao Zhu, Yuyang Zhang, Weiguo Hu, Xiaohong Xue, Zhiling Zhu, Libing Xiang, Jiarui Li, Xuhong Fang, Shujun Gao, Hua Feng, Wenjing Diao, Hongwei Zhang, Ming Du, Yongrui Bai, Yanli Hou, Weili Yan, Hao Feng, Hailing Yu, Shurong Zhu, Yan Du, Hua Jiang

**Affiliations:** 1 Obstetrics and Gynecology Hospital, Fudan University, Shanghai, 200011 China; 2 Shanghai Jiao Tong University School of Medicine, Renji Hospital, Shanghai, China; 3 Xinhua Hospital Affiliated to Shanghai Jiao Tong University School of Medicine, Shanghai, China; 4 Cancer Hospital of the University of Chinese Academy of Sciences (Zhejiang Cancer Hospital), Hangzhou, China; 5 The First Affiliated Hospital of Wenzhou Medical University, Wenzhou, China; 6 Zhongshan Hospital Affiliated to Fudan University, Shanghai, China; 7 Children’s Hospital of Fudan University, Shanghai, China

**Keywords:** Cervical cancer, Stages IB1, IB2, and IIA1, Laparoscopic radical hysterectomy, Abdominal radical hysterectomy, Randomized controlled trials, Progression-free survival, Overall survival, Prognosis

## Abstract

**Background:**

A retrospective study and a randomized controlled trial published in late 2018 have shown that laparoscopic radical hysterectomy (RH) was associated with worse survival than abdominal RH among patients with early-stage cervical cancer. Radical hysterectomy in cervical cancer has been a classic landmark surgery in gynecology; therefore, this conclusion is pivotal. The current trial is designed to reconfirm whether there is a difference between laparoscopic RH and abdominal RH in cervical cancer (stages IB1, IB2, and IIA1) patient survival under stringent operation standards and consistent surgical oncologic principles.

**Methods/design:**

This is an investigator-initiated, Prospective, Randomized, Open, Blinded End-point (PROBE)-controlled non-inferiority trial. A total of 780 patients with stage IB1, IB2, and IIA1 cervical cancer will be enrolled over a period of 3 years. Patients are randomized (1:1) to either the laparoscopic RH or the abdominal RH group. Patients will then be followed up for at least 5 years. The primary endpoint will be 5-year progression-free survival, and secondary endpoints include 5-year overall survival, recurrence, and quality of life measurements.

**Discussion:**

The debate on laparoscopic versus abdominal RH is still ongoing, and high-quality evidences are needed to guide clinical practice. The study results will provide more convincing evidence-based information for early-stage cervical cancer patients and their gynecologic cancer surgeons in their choice of surgical method.

**Trial registration:**

ClinicalTrials.govNCT04929769. Registered on 18 June 2021

## Background

Cervical cancer is one of the main malignant tumors that threaten the health and lives of women worldwide, and it is also the fourth leading cause of cancer-related deaths in women [1]. Radical cervical cancer surgery is the main procedure for the treatment of early-stage cervical cancer and is mainly applicable to cervical cancer above stage IA1 (including IA1 with LVSI, IA2, IB1, IB2, IIA1, IB3, and IIA2) [2]. Compared with standard total hysterectomy, radical hysterectomy for cervical cancer is more difficult to perform and may result in more complications due to a larger resection margin. With a history of more than 120 years, the surgical approach of abdominal radical hysterectomy for cervical cancer has been well developed. In addition, minimally invasive radical hysterectomy for cervical cancer has also become well established over the past two decades. Compared with traditional abdominal radical hysterectomy (ARH), minimally invasive radical hysterectomy has unique advantages; therefore, the 2014 NCCN guideline recommended laparoscopic radical hysterectomy (LRH) when performing radical cervical cancer surgery. A few small studies have evaluated the survival benefits of LRH for cervical cancer [3–8]. They reported that LRH was not inferior to ARH in progression-free survival (PFS), while laparoscopy was superior to open surgery in operative time, intraoperative bleeding, and length of hospital stay [3–8].

In November of 2018, the *New England Journal of Medicine* published two studies comparing abdominal and minimally invasive radical cervical cancer surgery [9, 10]. The retrospective cohort study concluded [9] that the 4-year mortality rate was significantly higher for women who underwent minimally invasive surgery than those who underwent open surgery. What is more, the randomized clinical trial (RCT), the Laparoscopic Approach to Cervical Cancer (LACC trial) [10] showed that the 4.5-year PFS rate was 86.0% in the minimally invasive surgery group and 96.5% in the open surgery group, with a difference of − 10.6 percentage points (95% CI − 16.4 to − 4.7). The minimally invasive surgery was also associated with lower disease-free survival (DFS) compared with open surgery [3-year DFS was 91.2% vs. 97.1%; hazard ratio for relapse or death of cervical cancer was 3.74; 95% CI (1.63 to 8.58)]. The results from the above two studies have caused a huge impact on the gynecology community. Following the publication of these studies, the version 3.2019 of the NCCN guidelines recommended open surgery as the standard procedure for cervical cancer, while laparoscopic surgery is no longer recommended.

However, after a careful review, we noticed issues in both studies. The retrospective cohort study could be hampered by confounders. There were significant differences in race and socioeconomic status between laparoscopic RH and abdominal RH groups. In the LACC trial, only 10 laparoscopic surgeries were required for the surgeon in the trial, which may not be adequate enough to achieve proficiency at laparoscopic RH. According to the trial protocol, the operation procedures may not meet the standard of type C radical hysterectomy by Querleu-Morrow (Q-M) classification, and surgical oncologic details were not mentioned. These issues may affect the results of this RCT.

Our hospital performs around 1500–2000 cervical cancer surgeries annually over the past 8 years. According to our data, the 5-year overall survival (OS) rate of laparoscopic radical cervical cancer surgery for stage IA2 and stage IB1 from 2014 to 2018 was 100% and 96.93%, respectively, which was similar to laparotomy in the LACC trial [10]. Moreover, our data shows that laparoscopy is superior to open surgery in both short-term complications and long-term survivals for endometrial cancer patients, in which laparoscopic surgery is the first choice recommended by the NCCN guidelines. So far, there is only one high-quality RCT (the LACC trial) comparing minimally invasive and open radical surgeries for cervical cancer. To have more conclusive evidence, we designed the current RCT to compare the outcomes of stage IB1, IB2, and IIA1 cervical cancer patients receiving laparoscopic RH vs. abdominal RH under consistent surgical oncologic operating regulations.

## Methods/design

### Trial design

**Fig. 1:**
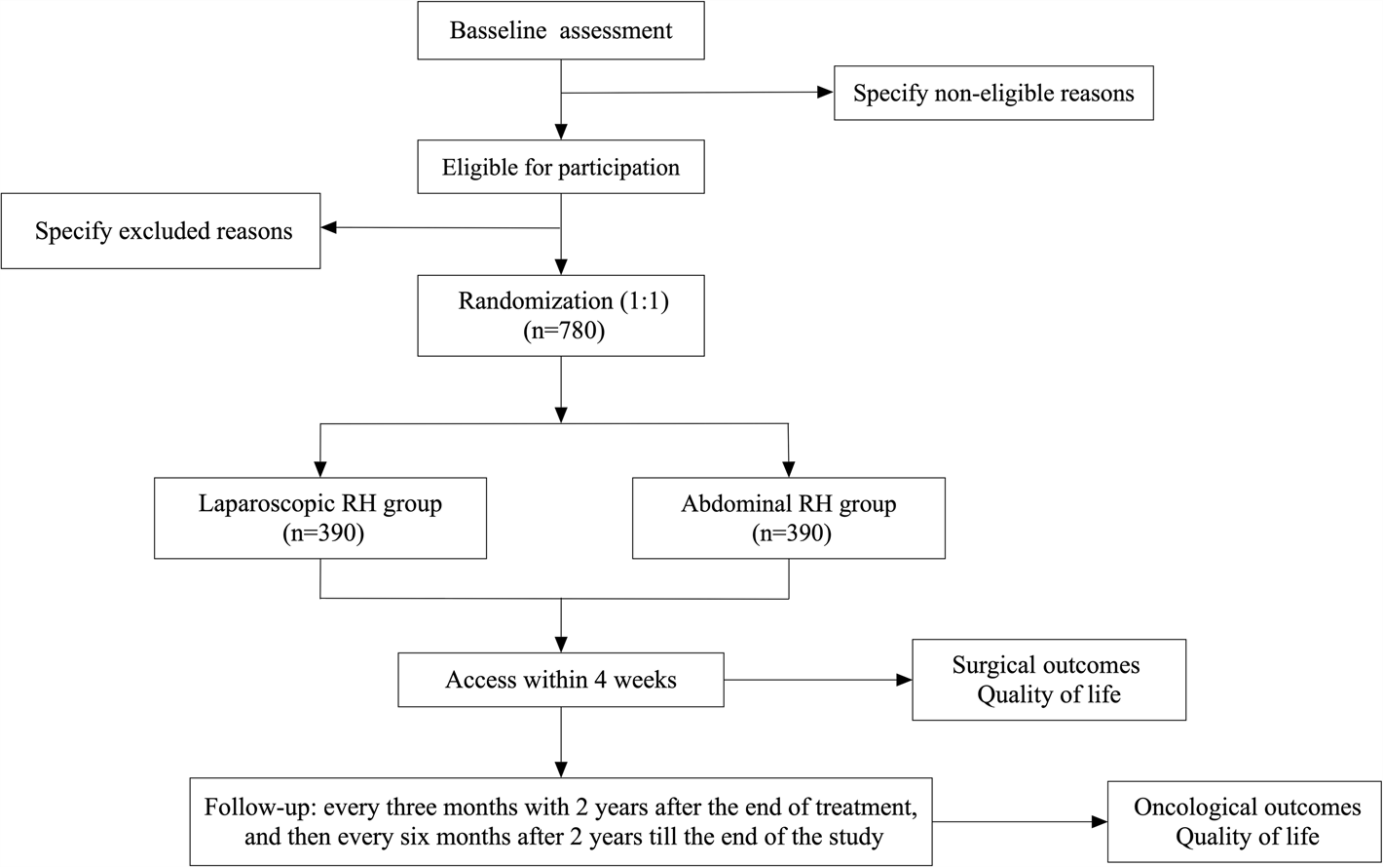
Study flow diagram

This research project is an investigator-initiated, Prospective, Randomized, Open, Blinded End-point (PROBE)-controlled non-inferiority trial. Patients are randomly assigned to receive abdominal RH or laparoscopic RH (Fig. 1). This study protocol adheres to the SPIRIT statement for clinical trial protocols [11] and the SPIRIT-PRO Extension [12].

A Trial Steering Committee (TSC) will be established for this study to review and oversee the study conduct and ensure patient safety, data collection, and analysis. The TSC is composed of the main center study leader, the sub-center leaders, and the study statistician. The steering committee will meet every 6 months to review the study progress and data quality, draft annual and final reports, and discuss any modifications if needed. Any modification of the trial protocol will be communicated to relevant parties by the principal investigator after agreement by the steering committee. It will provide oversight and relevant information for other committees. The Trial Coordinating Center is set up at the main center, which is composed of dedicated research staff and will provide day-to-day support for the trial. The coordinating center is responsible for scheduling periodic or emergent meetings, collecting and releasing information, and receiving feedback from the sub-centers, coordinating and handling temporary situations that arise during the study and ensuring the study quality is consistent across each center. An independent data monitoring committee (DMC) composed of a gynecologist, an epidemiologist, and a statistician is installed to oversee patients’ safety and the quality of the trial. The DMC met after recruitment had started to establish a charter and will hold formal meetings at least annually. Adverse events (AEs) are recorded and reported by the attending gynecologist according to the charter.

### Recruitment and eligibility

Potential participants will be patients with newly diagnosed stage IB3 and IIA2 cervical cancer at the Obstetrics and Gynecology Hospital of Fudan University, Zhongshan Hospital of Fudan University, Renji Hospital of Shanghai Jiao Tong University, Xinhua Hospital of Shanghai Jiao Tong University, Taizhou Hospital Affiliated Cancer Hospital of the University of Chinese Academy of Sciences, and First Affiliated Hospital of Wenzhou Medical University in China. The main center (Obstetrics and Gynecology Hospital, Fudan University) performs around 1500 cervical cancer surgeries (stages IB1, IB2, and IIA1) annually, and the other centers perform 100–300 (stages IB1, IB2, and IIA1) cervical cancer surgeries annually each. The sub-PIs from these hospitals are all experienced surgeons and have performed at least 800 cervical cancer operations. All of them have participated in national competitions, and three of them have won the championship.

The study centers will advertise this study at their cervical cancer clinic. The staging is confirmed after independent examination of the patients by two senior physicians based on the pathology report, as well as pelvic MRI/CT results. Dedicated research staff members will screen potential participants and approach them nicely. If the patients are interested, they will be further introduced in detail about the study background, randomization, and surgical procedures. The chief attending physician will enroll the patients. The enrolled patients will be given priority when scheduling follow-up and specific treatment.

#### Inclusion criteria

Participants meeting all of the following criteria will be considered for enrollment:

i. Clinical diagnosis of squamous carcinoma of the cervix, adenocarcinoma, and squamous adenocarcinoma (stages IB1, IB2, and IIA1)
ii. Age ≥ 21 years and ≤ 70 years
iii. Surgery type C (refer to Q-M surgical staging)
iv. Normal range of liver and kidney function and blood count (hemoglobin > 60 g/L, platelets > 70 × 10^9^/L, leukocytes > 3 × 10^9^/L, creatinine < 50 mg/dL, transaminase abnormal indicators ≤ 3, maximum value of transaminases not exceeding 3 times the corresponding normal value)
v. No history of other malignancies
vi. No pregnancy
vii. Physical strength classification: Karnofsky score ≥ 60
viii. Voluntarily join the study, sign the informed consent form, and compliant and cooperative with the follow-up
ix. No psychiatric disorders and other serious immune system disorders (e.g., lupus erythematosus, myasthenia gravis, HIV infection) (note: maximum diameter measurement of cervical lesions is based on PET-CT, CT, or MRI)

#### Exclusion criteria

Participants meeting one of the following criteria will be excluded from enrollment into this study:

i. Those who are contraindicated for various surgeries and cannot undergo surgery
ii. Patients who have received pelvic/abdominal radiotherapy irradiation or neoadjuvant chemotherapy for cervical cancer
iii. Recurrent cervical cancer patients with CT, MRI, or PET-CT suggesting suspicious metastasis of the pelvic lymph nodes with a maximum diameter > 2 cm after further preoperative examination

### Specific aims

We intend to conduct the current study to compare the outcomes of laparoscopic RH vs. abdominal RH in stage IB1, IB2, and IIA1 cervical cancer patients under a well-controlled setting. The *primary aim* is to test the hypothesis that the rate of PFS at 5 years with laparoscopic RH is not inferior to that of the abdominal RH. One of the secondary aims is to assess the differences in OS, intraoperative and perioperative complications, surgical indicators, and life quality measurements.

#### Primary endpoint

The primary endpoint will be 5-year PFS, defined according to the Response Evaluation Criteria in Solid Tumors (RECIST) criteria.

#### Secondary endpoints

The following are the secondary endpoints:

i. Five-year OS, which is defined as the time from surgery till death; patients who are alive at the last contact will be censored for OS.
ii. Operation time, anesthesia time, and intraoperative bleeding volume.
iii. Intraoperative complications and postoperative complications.
iv. Postoperative pain score, postoperative hospital stay, 1-month postoperative quality of life, 1-year postoperative quality of life, and sexual quality of life.

### Randomization, blinding, and treatment allocation

Before randomization, all eligible patients are required to provide written informed consent. A centralized block randomization with a block size of six will be used to randomly assign patients to ensure that there will be an equal number of patients (1:1) in the laparoscopic/robotic RH and abdominal RH groups. The randomization scheme will be completed by an independent statistical team at the Clinical Trial Center of the Children’s Hospital of Fudan University by creating and sequencing random seed numbers (SAS 9.4software). The intervention protocols determined by the random assignment sequence will be in sequentially numbered, non-transparent, sealed envelopes by zone: each of the six subgroups for each zone will be placed sequentially in six small, non-transparent, sealed envelopes numbered from 1 to 6 and then uniformly placed in the same large envelope marked with the corresponding zone number. The details of the allocation sequence are unknown to the investigators and surgeons. After completing the baseline measurements, the randomization assignment will be administered by the central coordinator at the Obstetrics and Gynecology Hospital of Fudan University who is not involved in the implementation of the intervention or the outcome observation. The enrollment is competitive, and the sub-center coordinator will contact the central coordinator before the inclusion of the first patient in each group. The central coordinator will follow the randomization scheme and assign the large envelopes with the group numbers to each center following the order of contact. Each sub-center will open the envelopes according to the order of signing the informed consent form, strictly follow the numbered order of the envelopes, and then inform the participating physicians of the assignment scheme and complete the written registration form. The surgeons will perform the operations according to the allocated random number.

Treatment allocation will not be blinded to the study personnel performing the surgery or patients. Patients will be informed about their group allocation after completion of the pre-surgery evaluation. Research staff evaluating the outcomes and data analysts will be blinded.

### Treatment

#### Accreditation of participating surgeons

Surgeons involved in this project were selected from each participating hospital and are skilled in cervical cancer surgery. To ensure trial quality, we set strict standards on the qualifications of the participating hospitals and surgeons. The lead surgeons should be proficient in both endoscopic and open surgeries and must have experience in at least 50 surgeries each for both LRH and ARH and be able to provide the medical record of these cases. The principal investigator and members of the surgical quality control team will be on site to observe each surgeon’s procedure and review the unedited video of the procedure (1 ARH and 1 LRH) provided by each surgeon for surgical quality measurement and documentation to ensure the extent of surgical resection and surgical oncologic management of each surgeon. All surgical procedures during the trial will be recorded and stored. The surgical quality control team will randomly select and review the unedited video and will give feedback to the corresponding surgeons. If there are any deviations during the surgical procedure, the quality control team will communicate with the surgeon and provide a specific monitoring period of 1 month for improvement and re-evaluation. In addition, a sensitive analysis will be performed during the statistical analysis phase.

#### Surgery therapy

Minimally invasive radical cervical cancer treatment includes laparoscopic radical cervical cancer treatment (LRH) and robotic-assisted laparoscopic radical cervical cancer treatment, either of which can be chosen by the surgeon. This project focuses on the survival benefit of patients after minimally invasive and open radical cervical cancer treatment, so radical cervical cancer treatment with preservation of reproductive function will not be included. For both open and minimally invasive radical cervical cancer surgery, the operation begins with a thorough abdominal exploration, including careful exploration of the diaphragm, and any metastatic lesions and metastatic sites should be described in detail in the operative record, with biopsy for confirmative diagnosis. If intra-abdominal lesions are found, radical cervical cancer surgery should not be used and should change to palliative treatment.

Pelvic lymph node dissection should be performed during radical cervical cancer surgery. Sentinel lymph node (SLN) mapping is not included in this project, since there is no sufficient evidence for its sensitivity and specificity, and also the difficulty in achieving lymph node hyperstaging considering the large sample size of this project. If the tumor is ≥ 2 cm, if the common iliac lymph node is positive for intraoperative freezing (optional), or if the preoperative evaluation of the paraaortic lymph nodes is positive, paraaortic lymph node dissection is preferred and followed by paraaortic lymph node biopsy. When clearing the paraaortic lymph nodes, it is enough for the upper border to reach the level of the inferior mesenteric artery. The common iliac lymph nodes should be sent separately for examination, and the resection should include both sides of the common iliac vessels, with the upper border reaching the midpoint of the bifurcation of the abdominal aorta and the common iliac vessels and the lower border reaching the level of the bifurcation of the common iliac vessels.

If definite pelvic lymph node metastasis is found intraoperatively, continuation of pelvic lymph node dissection and even radical cervical cancer surgery is not required, but abdominal paraaortic lymph node sampling is recommended to assess the degree of disease progression and to develop subsequent radiotherapy regimens. If surgery is continued, radical cervical cancer surgery + pelvic lymph node dissection + abdominal paraaortic lymph node dissection/biopsy is recommended.

For stages IB1, IB2, and IIA1, the surgical approach is type C1 (radical cervical surgery with preservation of autonomic nerves + pelvic lymph node dissection, recommended for tumor diameter < 2 cm) or type C2 (radical cervical surgery with bilateral pelvic lymph node dissection, resection of 3–4 cm of the parametrium and the upper 1/4 to 1/3 of the vagina) with abdominal paraaortic lymph node dissection if necessary (surgical staging according to FIGO 2018, staging according to Q-M staging). The depth of parametrial tissue removed should be below the deep uterine vein, and if preoperative involvement of the vaginal wall is considered, 1–2 cm of parametrial tissue should be removed.

Implementing laparoscopic radical hysterectomy or abdominal hysterectomy will not require alteration to usual care pathways (including the use of any medication), and these will continue for both trial arms.

#### Surgical oncologic principles

In order to avoid the dissemination or recurrence of tumors during the operation, we have composed detailed regulations for tumor-free operation, especially for laparoscopic surgery.

i. Encourage sharp resection of the diseased area during the operation and try to avoid strong tearing, pulling, and other processes.
ii. Instead of using a uterine lifting device, use silk thread to pull the uterine body to assist the exposure of the surgical field of view.
iii. Entire resection of the lymph node without touching other parts; entire resection of the parauterine tissue that needs to be removed from the extensive uterus, avoiding excessive traction during the operation.
iv. After closing the vagina with kidney pedicle forceps, cut the anterior wall of the vagina, insert the gauze into the vagina, cut off the posterior wall, and disinfect the vaginal stump with an alcohol cotton ball.
v. Devices that have been in contact with tumors in the vagina are separated from other devices and will not be used during the operation.
vi. After the pelvic and abdominal cavity is fully rinsed with sterilized water after the operation, the residual water in the pelvic cavity/abdominal cavity should be absorbed as clean as possible.

For laparoscopic surgery, the following points should be emphasized:

i. During the operation, the pelvic lavage fluid should be prevented from pouring into the upper abdomen when the head is lowered and the feet are high.
ii. Before cutting off the vagina, the vagina should be closed. The methods that can be used include: closing the vagina with a closure device, closing the vagina with a ligation ring, and suturing the vagina through the vagina.
iii. After the vagina is closed under the laparoscope, it should be washed with sterile water and be disconnected, and then the vagina should be sutured. It should be ensured that the cut vagina does not touch the diseased part.
iv. During the operation, the whole lymph node should be removed from the vagina after the uterus is disconnected without touching the other parts and directly put into the specimen bag to be removed vaginally after the uterus is severed.
v. If suspicious metastasis in the pelvic or abdominal cavity is detected during the operation, do not touch the other parts after the resection, directly put it into the specimen bag, and take it out through the vagina.

### Follow-up protocol

Follow-up will be conducted at the dedicated unit at each center. Written informed consent should be obtained before any protocol-related procedures. See Table 1 for the study procedures and baseline and/or follow-up assessments.

**Table 1:** Study procedures and baseline and/or follow-up assessments

Visit number	Screening for eligibility	Randomization	Blood examination, urinary test, liver and renal function, ECG	Surgical indicators, surgical complications	Gynecological examination	Imaging test	SCCA/CA-125	Cervical liquid-based cytology	HPV test	Adverse events	EORTC QLQ-C30 v3.0; EORTC QLQ-CX24
V0	×										
V1		×	×	×	×	×	×	×	×	×	
V2 (3 months)					×	×	×	×		×	×
V3 (6 months)					×	×	×	×	×	×	×
V4 (9 months)					×	×	×	×		×	×
V5 (1 year)					×	×	×	×	×	×	×
V6 (15 months)					×	×	×	×		×	×
V7 (1.5 years)					×	×	×	×		×	×
V8 (21 months)					×	×	×	×		×	×
V9 (2 years)					×	×	×	×		×	×
V10 (2.5 years)					×	×	×	×	×	×	×
V11 (3 years)					×	×	×	×	×	×	×
V12 (3.5 years)					×	×	×	×	×	×	×
V13 (4 years)					×	×	×	×	×	×	×
V14 (4.5 years)					×	×	×	×	×	×	×
V15 (5 years)					×	×	×	×	×	×	×

*ECG* electrocardiogram, *EORTC* European Organization for Research and Treatment of Cancer, *QLQ* Quality of Life Questionnaire, *SCCA* SCC antigen test

At visit 0 (V0), patients are screened for eligibility, and eligible patients are scheduled for enrollment. At the randomization (V1), patients are randomly assigned to either the laparoscopic RH group or the abdominal RH group. Before surgery, the following items will be tested and recorded: routine blood examination, urinary test, liver and renal function, ECG, gynecological examination, SCC antigen test (SCCA) if squamous cell carcinoma/CA-125 if adenocarcinoma, HPV testing, cervical liquid-based cytology, imaging test (chest CT + pelvic enhanced MRI + epigastric enhanced MRI, or PET-CT + pelvic enhanced MRI), the sonographic results (ultrasound of the liver, gall, pancreas, kidney, and ureter), and pathology results of tissue biopsy or LEEP results for patients of IA1 with LVSI and IA2. The following intraoperative and postoperative information will be recorded: imaging test to evaluate retroperitoneal lymph node metastasis, details of the operation, surgical indicators such as operation time and intraoperative blood loss, duration of anesthesia, postoperative pain scores and surgical complications, and other special circumstances.

Clinically, follow-up visits will be conducted every 3 months within 2 years after the surgery and then every 6 months after 2 years till the end of the study (Fig. 1). Patients will be sent a text message 2 weeks before each follow-up visit, and a special made booklet containing follow-up test results will be recorded and kept by the investigators. At the V2–V9 visits, the following evaluation will be performed and results be recorded: imaging test (chest CT + pelvic enhanced MRI + epigastric enhanced MRI, or PET-CT + pelvic enhanced MRI), gynecological examination, SCCA if squamous cell carcinoma/CA-125 if adenocarcinoma, cervical liquid-based cytology, HPV test (only at V3 and V5), adverse events, the European Organization for Research and Treatment of Cancer (EORTC) Quality of Life Questionnaire - Core 30 (QLQ-C30) version 3.0 to evaluate the quality of life, and EORTC Quality of Life Questionnaire Cervical Cancer Module (QLQ-CX24) to evaluate the cervical cancer-specific quality of life. At the V10–V15 visits, the following evaluation will be performed and results be recorded: gynecological examination, SCCA if squamous cell carcinoma/CA-125 if adenocarcinoma, cervical liquid-based cytology test, high-risk HPV test, imaging test (chest CT + pelvic enhanced MRI + epigastric enhanced MRI, or PET-CT + pelvic enhanced MRI), adverse events, life quality (EORTC QLQ-C30 v3.0), and sex life quality (EORTC QLQ-CX24).

To promote participant retention and complete follow-up, the study team members at each center will give detailed patient orientation during the recruitment phase. The study team members will provide long-term remote condition assessment services and related information consultation services for all study participants. Patients will be followed up regularly by dedicated staff to reduce the rate of loss to follow-up. The follow-up system will regularly remind the patients of the follow-up time and relevant items according to the follow-up protocol. In case of patient withdrawal, the investigator will make appropriate efforts to determine and document the reasons and obtain the patient’s consent to continue monitoring their disease status (relapse, survival, toxicity, etc.) through the patient’s medical record. If a patient moves to another study treatment and requires changing physician, the investigator will make a reasonable attempt to locate that center’s physician and request assistance in order to complete the follow-up.

### Statistical methods

This study uses a non-inferiority trial design, and the primary outcome is the 5-year PFS rate. The sample size is calculated based on the difference between the two groups of 5-year PFS rate. The 5-year OS rate of the patients in the open surgery group is estimated to be 92% based on clinical data from our hospital, and the 5-year PFS rate is lacking. To infer the relationship between the 5-year PFS rate and the 5-year OS rate in patients undergoing open surgery, we refer to previous studies and construct a linear regression equation with the 5-year OS rate as the independent variable *X* and the 5-year PFS rate as the dependent variable *Y* (*Y* = 1.199 *X*-0.219). Therefore, the 5-year PFS rate of the open surgery group is estimated to be 88%, when the 5-year OS rate is 92%. Based on the assumptions of (i) a 5-year PFS rate of 88% in the open surgery group, (ii) a non-inferiority margin of 7%, (iii) 80% power, and (iv) a one-sided alpha of 0.025, the sample size estimation resulted in 350 subjects per group (calculated with the SAS software, version 9.4). Considering a 10% drop-out rate and the randomization scheme with a block size of 6, a total of 780 subjects should be randomized (390 per group).

All analyses will be performed on an intention-to-treat (ITT) basis, that is, by treatment received including all randomized patients. Missing data will be censored at the date they are last known to be alive. Sensitivity analysis will be performed according to the per-protocol (PP) treatment, which only includes those patients who are treated according to the protocol. All statistical analyses will be performed with a two-sided significance level of 0.05 and conducted using the SAS software, version 9.4 (SAS Institute).

#### Analysis of primary outcome data

The curves of PFS at 5 years will be estimated using the Kaplan-Meier method. The log-rank test will be used to test the above hypothesis, and the 5-year PFS rate difference and its 95% confidence interval (CI) for the comparison between the two groups will be estimated. The minimally invasive surgery will be considered non-inferior to the open surgery if the one-sided 95% upper limit is less than, where the predetermined non-inferiority margin = 6%. An analysis of the primary outcome adjusting for the blood loss during operation, operative duration, and postoperative pain score will be performed using the Cox proportional hazards regression model. The hazard ratio of 5-year PFS and corresponding 95% CI will be estimated. The stratified analysis will be performed according to tumor stage, and Cox proportional hazards regression model will be used to estimate the hazard ratio and corresponding 95% CI of the 5-year PFS.

#### Analysis of secondary outcome data

Continuous outcomes include operative duration, anesthesia time, blood loss during operation, postoperative pain score, and postoperative hospital stay. The outcomes with normal distribution will be summarized using mean and standard deviation (SD), while the outcomes with non-normal distribution will be summarized using median and interquartile. The differences in the outcomes and 95% CIs will be analyzed by a generalized linear model (GLM) with treatment as a fixed effect and with normal distribution and identity link function.

The intraoperative complications, postoperative complications, 1-month and 1-year postoperative quality of life, and sexual life will be treated as binary outcomes and summarized by the number (%) of participants with the event. The differences in the outcomes and 95% CIs will be analyzed by GLM with treatment as a fixed effect and with binomial distribution and identity link function.

Adverse events (AEs) will be summarized using the number of AEs and the number (%) of participants with AEs by groups. The interim analyses will be performed 3 months postoperatively after 50, 100, 150, 200, and 300 patients are randomized into each group. All AE data will be reviewed and assessed by the Data Monitoring Committee which will make recommendations to the Trial Steering Committee if the protocol needs revision or the trial should be stopped.

### Data confidentiality and storage

The research assistant will collect patient personal information and medical information from medical files after the participants’ consent. All data will be quality checked and double entered into a secure electronic database. Paper and electronic data will be stored at a designated research office in the hospital, which can only be accessed by the research staff. Paper copy data will be securely held in a locked filing cabinet, and electronic data will be stored on a password-protected computer. Follow-up information will be linked to the baseline clinicopathological database using the unique patient ID number. All identifiable data will be removed for analysis to protect the patients’ privacy.

### Ethical considerations

The study was approved by the Institutional Review Board of the Obstetrics and Gynecology Hospital of Fudan University in Shanghai, China (Reference number: 2021-04; date of approval: 18 January 2021). This study is conducted in accordance with the Declaration of Helsinki.

All patients will be given both written and oral information about the study from their gynecologist during admission for surgery. Patients must sign an informed consent form in accordance with the Declaration of Helsinki before being included in the study. Refusal to participate will not affect the standard treatment. Patients can withdraw from the study at any time during the study period. The patients or their family members can address their concerns or queries about the study-related questions to the project leaders (HJ or XW) throughout the study period. The investigator has the right to withdraw a patient from the trial treatment or study in the event of secondary diseases, adverse events, protocol violations, administrative reasons, or other reasons. There is no anticipated harm and compensation for trial participation; therefore, no provision for post-trial care is planned.

Patients or the public are not involved in the design, conduct, reporting, or dissemination plans of this research. Study results will be submitted for publication in international medical journals and on the hospital website and presented at conferences.

## Discussion

Although the incidence of cervical cancer has been declining in developed countries such as the USA, Europe, Australia, and New Zealand due to high compliance with Pap smear screening programs and HPV vaccination, it still remains one of the main gynecologic malignancies in developing countries including China [13]. So far, China has the largest population in the world. Therefore, it is crucial to choose both clinically and culturally acceptable as well as cost-effective treatment strategies.

Influenced by the Chinese culture, a large proportion of cervical cancer patients prefer complete tumor resection when making treatment choices. In addition, there is an increasing number of young patients, who favor smaller surgical incisions and faster recovery. At the same time, the rapid development and refinement of laparoscopic techniques have become more prominent in the past decade, and well-trained Chinese surgeons have also made continuous efforts in refining the endoscopic technique and surgical oncologic procedure. Until now, a set of standard surgical procedures and steps has been developed for laparotomy.

Most surgeons follow certain specifications when learning surgical skills. Practice makes perfect. However, the application and development period of laparoscopic surgery is relatively short, and a specific and unified process has not yet been formed. A series of procedures may cause the spread of tumors. For example, surgeons can choose to perform lymph node dissection or excision of enlarged lymph nodes or separation of parauterine tissues first based on individual preference. Some surgeons would use uterine manipulator during the operation, and the vagina is not closed before the uterus is cut, or the vagina is not washed when the uterus is removed. In addition, different surgeons have variations in terms of complete resection and surgical oncologic principles, which can also affect the results. To reduce the interference of these factors, the current trial has set stringent and consistent surgical criteria, especially for laparoscopic surgery. Besides individual variation, there are also differences among hospitals. Therefore, the current trial set up a technical team to inspect the surgical equipment of the sub-centers and monitor the surgical qualifications of the surgeons, so as to control the surgical quality of the surgeries and minimize the impact of technical issues on the outcomes.

In this trial, we selected patients with stages IB1, IB2, and IIA2, since those patients account for most of the cervical cancer. Tumor size at these stages is smaller than 4 cm, and the type C1 surgical method is recommended according to the NCCN guidelines, which facilitates further comparisons of the two surgical methods. So far, there is only one high-quality RCT (the LACC trial) that evaluated the long-term survival of minimally invasive and open radical surgeries for early-stage cervical cancer patients. The debate on laparoscopic versus abdominal RH is still ongoing, and high-quality evidences are needed to guide clinical practice. In this randomized trial, we aim to evaluate whether laparoscopic is not inferior to abdominal radical hysterectomy for patients with cervical cancer stages IB1, IB2, and IIA1 through detailed technical improvement. The study results will provide more convincing evidence-based information for early-stage cervical cancer patients and their gynecologic cancer surgeons in their choice of surgical method.

## Trial status

The study was approved by the Institutional Review Board of the Obstetrics and Gynecology Hospital of Fudan University in Shanghai, China on 18 January 2021. The trial was registered on ClinicalTrials.gov, and the registration number was obtained on 18 June 2021. Recruitment of participants started in May 2021 and is estimated to finish in May 2024. The last participant is expected to reach the primary endpoint (5-year follow-up) in May 2029. Primary data analysis will begin in May 2026. The protocol version number and date were 1.0 and 21 December 2020, respectively.

## Data Availability

The full protocol could be downloaded at ClinicalTrials.gov (NCT04929769, https://www.clinicaltrials.gov/ct2/show/NCT04929769?term=NCT04929769&draw=2&rank=1). Data generated during this study and statistical code are available upon reasonable request from the study PI after review by the Trial Steering Committee.

## Abbreviations

DFS: Disease-free survival
DMC: Data monitoring committee
ECG: Electrocardiogram
EORTC: European Organization for Research and Treatment of Cancer
FIGO: International Federation of Gynecology and Obstetrics
HR: Hazard ratios
ITT: Intention-to-treat
NCCN: National Comprehensive Cancer Network
OS: Overall survival
PFS: Progression-free survival
PP: Per-protocol
PROBE: Prospective, Randomized, Open, Blinded End-point
QLQ: Quality of Life Questionnaire
Q-M: Querleu-Morrow
RCT: Randomized clinical trial
RH: Radical hysterectomy
SCCA: SCC antigen test
95%CI: 95% confidence interval
